# German Health Update Fokus (GEDA Fokus) among Residents with Croatian, Italian, Polish, Syrian, or Turkish Citizenship in Germany: Protocol for a Multilingual Mixed-Mode Interview Survey

**DOI:** 10.2196/43503

**Published:** 2023-04-12

**Authors:** Carmen Koschollek, Marie-Luise Zeisler, Robin A Houben, Julia Geerlings, Katja Kajikhina, Marleen Bug, Miriam Blume, Robert Hoffmann, Marcel Hintze, Ronny Kuhnert, Antje Gößwald, Patrick Schmich, Claudia Hövener

**Affiliations:** 1 Department for Epidemiology and Health Monitoring Robert Koch Institute Berlin Germany; 2 Department for Infectious Disease Epidemiology Robert Koch Institute Berlin Germany

**Keywords:** migration, interview survey, hard-to-survey, health inequalities, mixed-mode, multilingual

## Abstract

**Background:**

Germany has a long history of migration. In 2020, more than 1 person in every 4 people had a statistically defined, so-called migration background in Germany, meaning that the person or at least one of their parents was born with a citizenship other than German citizenship. People with a history of migration are not represented proportionately to the population within public health monitoring at the Robert Koch Institute, thus impeding differentiated analyses of migration and health. To develop strategies for improving the inclusion of people with a history of migration in health surveys, we conducted a feasibility study in 2018. The lessons learned were implemented in the health interview survey German Health Update (Gesundheit in Deutschland aktuell [GEDA]) Fokus, which was conducted among people with selected citizenships representing the major migrant groups in Germany.

**Objective:**

GEDA Fokus aimed to collect comprehensive data on the health status and social, migration-related, and structural factors among people with selected citizenships to enable differentiated explanations of the associations between migration-related aspects and their impact on migrant health.

**Methods:**

GEDA Fokus is an interview survey among people with Croatian, Italian, Polish, Syrian, or Turkish citizenship living in Germany aged 18-79 years, with a targeted sample size of 1200 participants per group. The gross sample of 33,436 people was drawn from the residents’ registration offices of 99 German municipalities based on citizenship. Sequentially, multiple modes of administration were offered. The questionnaire was available for self-administration (web-based and paper-based); in larger municipalities, personal or phone interviews were possible later on. Study documents and the questionnaire were bilingual—in German and the respective translation language depending on the citizenship. Data were collected from November 2021 to May 2022.

**Results:**

Overall, 6038 respondents participated in the survey, of whom 2983 (49.4%) were female. The median age was 39 years; the median duration of residence in Germany was 10 years, with 19.69% (1189/6038) of the sample being born in Germany. The overall response rate was 18.4% (American Association for Public Opinion Research [AAPOR] response rate 1) and was 6.8% higher in the municipalities where personal interviews were offered (19.3% vs 12.5%). Overall, 78.12% (4717/6038) of the participants self-administered the questionnaire, whereas 21.88% (1321/6038) took part in personal interviews. In total, 41.85% (2527/6038) of the participants answered the questionnaire in the German language only, 16.69% (1008/6038) exclusively used the translation.

**Conclusions:**

Offering different modes of administration, as well as multiple study languages, enabled us to recruit a heterogeneous sample of people with a history of migration. The data collected will allow differentiated analyses of the role and interplay of migration-related and social determinants of health and their impact on the health status of people with selected citizenships.

**International Registered Report Identifier (IRRID):**

DERR1-10.2196/43503

## Introduction

### Migration in Germany

Germany is a country of immigration and was the second top destination country worldwide after the United States in 2019 [1]. In 2020, 27% of the population had a so-called migration background, which is defined by the Federal Office for Statistics as the person themselves or at least 1 of their parents were born with a citizenship other than German [2]. In Germany, 17% of the population had their own history of migration [2]. One of the relevant events in the recent history of immigration to Germany was the signing of multiple so-called guest workers recruitment contracts with countries in Southern Europe such as Turkey and Italy and countries in North Africa from the 1950s onward, followed by many returns but also by the immigration of some of the workers’ families. Since the 1950s, the immigration of the so-called (late) repatriates from countries of the former Soviet Union began, with most people immigrating during the 1980s and 1990s. During the 1990s, people from the former Yugoslavia fled the wars, and since the 2000s, people from Eastern European countries, for example, Poland, Romania, and Bulgaria, immigrated to Germany or commuted between Germany and their countries of origin in accordance with the European Union Freedom of Movement Act. In particular, during the 2010s, refugees came to Germany from countries such as Iraq, Afghanistan, or Syria. All of these different and heterogeneous groups of migrants and their descendants are subsumed under the statistical category of people with a migration background—except for the so-called (late) repatriates—although their duration of residence, reasons for migration, experiences during the migration process, residence status, and living situations differ. Hence, to identify health resources and health risks in epidemiological research on migration and health, multiple determinants that go beyond the category of migration background need to be considered.

### Migration and Health

Previous research showed specific differences in health outcomes between the general population and people with a history of migration in Germany. For example, higher rates of chronic preconditions or diseases, such as vascular and heart disease, obesity, and diabetes, are observed in people with a history of migration [3]; the prevalence of diabetes (mellitus and gestational) is found to be higher in first-generation migrants from Turkey [4]. Furthermore, the prevalence of specific infectious diseases, such as tuberculosis, is higher in migrant groups from endemic areas (eg, several Eastern European and Central Asian countries) [5,6]. Lower outcomes regarding psychological well-being were reported for asylum seekers and refugees than for the general population [7]. In addition, differences were observed in the utilization of health care services, for example, in terms of specialist health care, medication use, or therapist consultation, which are utilized less often by people with a migration background than by those without a migration background [8]. Moreover, the rates of utilization of preventive services, such as cancer screening or health checkups [9], as well as rehabilitative care services [10], are lower among people with a migration background.

The mechanisms underlying these differences in health outcomes cannot be explained by solely focusing on the category of migration background. Social determinants of health—as in the general population as well—need to be considered, for example, living situation; income; the risk of poverty; working conditions [10]; the impact of perceived discrimination [11,12]; or structural barriers in accessing health care services, which are especially prevalent among asylum seekers and refugees [13-15]. To facilitate such analyses, it is essential to include people with a history of migration into national public health monitoring, namely (1) equivalent to their proportion of the population and (2) considering their heterogeneity, for example, by offering different modes of survey administration or questionnaire languages to satisfy different needs.

### Participation of People With a History of Migration in Interview Surveys

During recent years, the participation of people with a history of migration was low in the “German Health Update” (Gesundheit in Deutschland aktuell [GEDA]), which is 1 of 3 parts of the national public health monitoring in Germany conducted by the Robert Koch Institute (RKI). For example, in GEDA 2019/2020, overall, 9% of the participants reported a country of birth other than Germany compared with 18.4% of the adult population whose country of birth is not Germany according to population statistics, and 3.9% reported a citizenship other than German compared to the 12.5% of adults living in Germany who have a citizenship other than German [16]. Thus, analyses of differences in health outcomes are difficult, as a selection bias among people with different countries of birth or citizenship might be assumed. Other studies focused on specific migrant groups, for example, those of Turkish origin [4,17,18], so-called (late) repatriates [19,20], or refugees [13,15,21]. Migration-related factors influencing health status can be analyzed within these groups; however, analyses across these groups are mostly not possible, as sampling procedures, methods of data collection, and survey indicators might differ. Moreover, comparability with the data from population-based public health monitoring is limited.

To overcome these issues, the project “Improving Health Monitoring in Migrant Populations” (IMIRA) was initiated at the RKI in 2016 [22]. One of its aims was to develop strategies for improving the inclusion of people with a history of migration in interview surveys. Therefore, a multilingual mixed-mode feasibility study was conducted among people with Croatian, Polish, Romanian, Syrian, or Turkish citizenship in 2 German federal states, namely Berlin and Brandenburg [23,24]. The findings and lessons learned from this feasibility study were subsequently implemented in the interview survey GEDA Fokus.

### Objectives of GEDA Fokus

As described, people with a history of migration are often underrepresented in the RKI health interview surveys (GEDA). Therefore, with GEDA Fokus, we aimed to conduct an interview survey among people with selected citizenships to provide comprehensive data on specific migrant groups to facilitate the analyses of relevant factors impacting their health status. The objectives of GEDA Fokus were as follows:

1. Description of the health status of the participants stratified by migration-related factors: the health status of the participants will be described based on a set of core indicators [25], considering migration-related factors with assumed impact on their health, such as duration of residence, residence status, motives of migration, German language proficiency, or perceived discrimination.
2. Methodological analyses to improve the response rate of people with a history of migration in future public health monitoring: the heterogeneity within people with a history of migration in terms of their level of education or income, German language proficiency, or trust in institutions makes differentiated approaches necessary for enabling the accessibility of (interview) surveys for different groups of people. Detailed analyses of recruitment strategies and the use of modes of administration can contribute to a better understanding of how specific subgroups can be reached in future interview surveys.

## Methods

### Study Design

The interview survey GEDA Fokus was a multilingual survey (Arabic, Croatian, Italian, German, Polish, and Turkish) applying a sequential mixed-mode design, offering a self-administered questionnaire on the web and, in the case of initial nonresponse, on paper. In larger municipalities, personal interviews by (partly) multilingual interviewers to be held either in the study persons’ homes or by telephone were also offered. The study persons were randomly selected from residents’ registration offices.

### Selection of the Study Population

Registries of residents’ registration offices were used for sampling, which provides a solid sampling frame for population surveys. Citizenship is the only characteristic captured here that can approximate migration history. By selecting people in this manner, naturalized persons or those of the second migrant generation with only German citizenship cannot be identified. To determine which citizenships to include in the survey (by citizenship), we used data from the Central Register of Foreigners from 2015 to 2017. We conducted model calculations using the Foreigners’ Statistic, which is based on the Central Register of Foreigners, considering the current stock, as well as the dynamics (inward and outward migration) of the top 10 citizenships according to the Foreigners’ Statistic in 2017. The most common citizenships identified over time in this manner were Italian, Polish, Romanian, Syrian, and Turkish; however, our feasibility study [23,24] showed that sampling from residents’ registration offices did not seem effective enough to reach people with Romanian citizenship because of high rates of ineligible addresses. Therefore, it was decided in accordance with the views of the members of the projects’ advisory board that Croatian citizenship be included instead of Romanian citizenship, as it was the next most relevant citizenship according to the model calculations. Thus, our study population included people with a registered Croatian, Italian, Polish, Syrian, or Turkish citizenship. Within residents’ registration offices, up to 3 citizenships of residents are captured, and all were considered.

### Selection of the Primary Sampling Units

**Figure 1:**
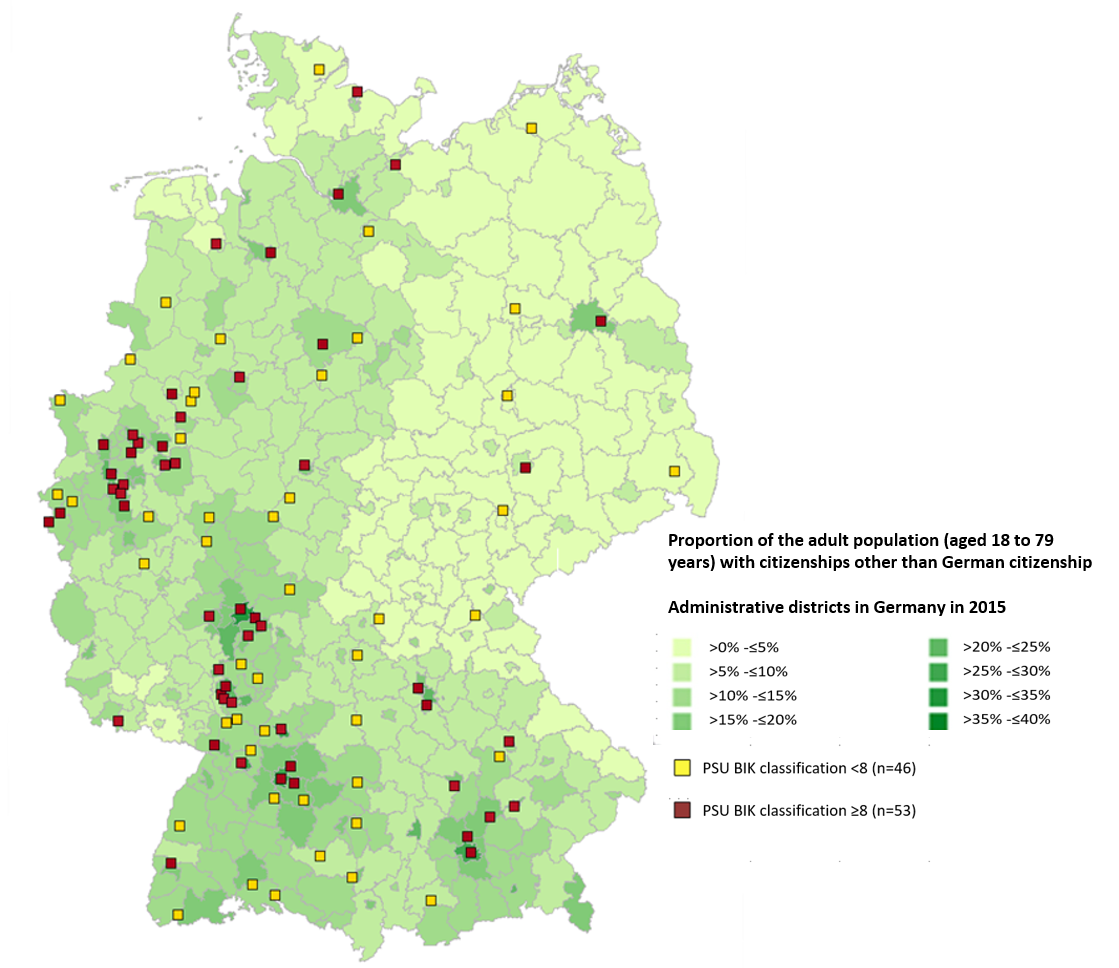
Primary sampling units (PSUs) of the interview survey German Health Update Fokus (GEDA Fokus). BIK: Beratung Information Kommunikation.

Primary sampling units (PSUs) were selected by colleagues at the GESIS Leibniz Institute for the Social Sciences using a Cox algorithm. This selection was based on the following stratification: the classification of federal states into regions (north, south, east, and west), the number of districts and urban municipalities per federal state, and the average proportion of the population without German citizenship within the districts and urban municipalities per federal state. Overall, 120 PSUs were selected (crude number of PSUs=99, as bigger cities such as Berlin were selected more than once): 74 with a Beratung Information Kommunikation (BIK) classification of ≥8 (urban PSUs; crude n=53) and 46 PSUs with a BIK classification of <8 (merely rural areas and smaller cities). The BIK classification systematically structures areas by describing relations between cities and surrounding areas in Germany [26]. A BIK classification of 8 is ascribed to the core areas of cities with 100,000 to 500,000 inhabitants, and classifications of 9 and 10 are ascribed to the core and surrounding areas of cities with ≥500,000 inhabitants [26]. The PSUs selected for GEDA Fokus are displayed in Figure 1.

### Participants and Inclusion Criteria

People were included in GEDA Fokus if they:

- had a Croatian, Italian, Polish, Syrian, or Turkish citizenship (first, second, or third citizenship according to residents’ registration office),
- were aged between 18 and 79 years,
- had their principal residence in one of the selected PSUs, and
- were able to give written informed consent for survey participation.

People were excluded from the survey if:

- their language proficiency in German or one of the offered translation languages was insufficient to answer the questionnaire or interview or
- they were not able to give written informed consent for survey participation.

### Sampling Procedures

Sampling was carried out at the RKI in 2 tranches; data were cleaned upon reception from the residents’ registration offices and forwarded via a secured data link to the external service provider “Kantar GmbH,” who conducted the data collection on behalf of the RKI. Kantar GmbH is a market research company that conducts social science and health research surveys. Tranche 1 included 15 PSUs with a BIK classification of <8 and 30 PSUs with a BIK classification of ≥8 (crude n=17). Tranche 2 included 31 PSUs with a BIK classification of <8 and 44 PSUs with a BIK classification of ≥8 (crude n=36). The gross sample comprised 39.53% (13,216/33,436) of study persons in tranche 1 and 60.47% (20,220/33,436) of study persons in tranche 2.

### Recruitment of the Participants

In the first study phase, all the selected study persons (N=33,436) received an invitation to participate by postal mail, including a link and log-in information to the web-based questionnaire. The invitation letter and the study information leaflet were bilingual, with a German version on one side and the respective translated version on the other. In the web-based survey, the participants were able to select the German language–only or a bilingual version (eg, German-Italian) of the questionnaire. To ask questions regarding the survey itself or to refuse participation, the study persons were offered contact options (hotline or email address). In addition, frequently asked questions were posted on a multilingual website.

In the second study phase, all the study persons who neither took part nor refused participation received postal mail with a bilingual reminder including the web link and log-in information for the web-based survey, a study information leaflet, a data privacy statement, and a bilingual paper-based questionnaire. The paper-based questionnaire featured a line-by-line translation (eg, German-Italian). At the end of the paper-based questionnaire, a bilingual consent form was attached that the study persons needed to sign and send back.

The third study phase varied between PSUs with a BIK classification of <8 and those with a BIK classification of ≥8. Study persons in the smaller PSUs only received a second bilingual reminder letter in the form of an enveloped postcard including the web link and log-in information for the web-based questionnaire. In the PSUs with a BIK classification of ≥8, a home visit was announced with the purpose of conducting a personal interview.

In the last study phase, the interviewers (partly bilingual) visited the study persons who had not taken part or refused participation at their homes to either conduct a personal interview or, depending on the preference of the study persons, obtain their telephone number and conduct a telephone interview afterward. The latter option was particularly offered to account for the SARS-CoV-2 pandemic.

**Figure 2:**
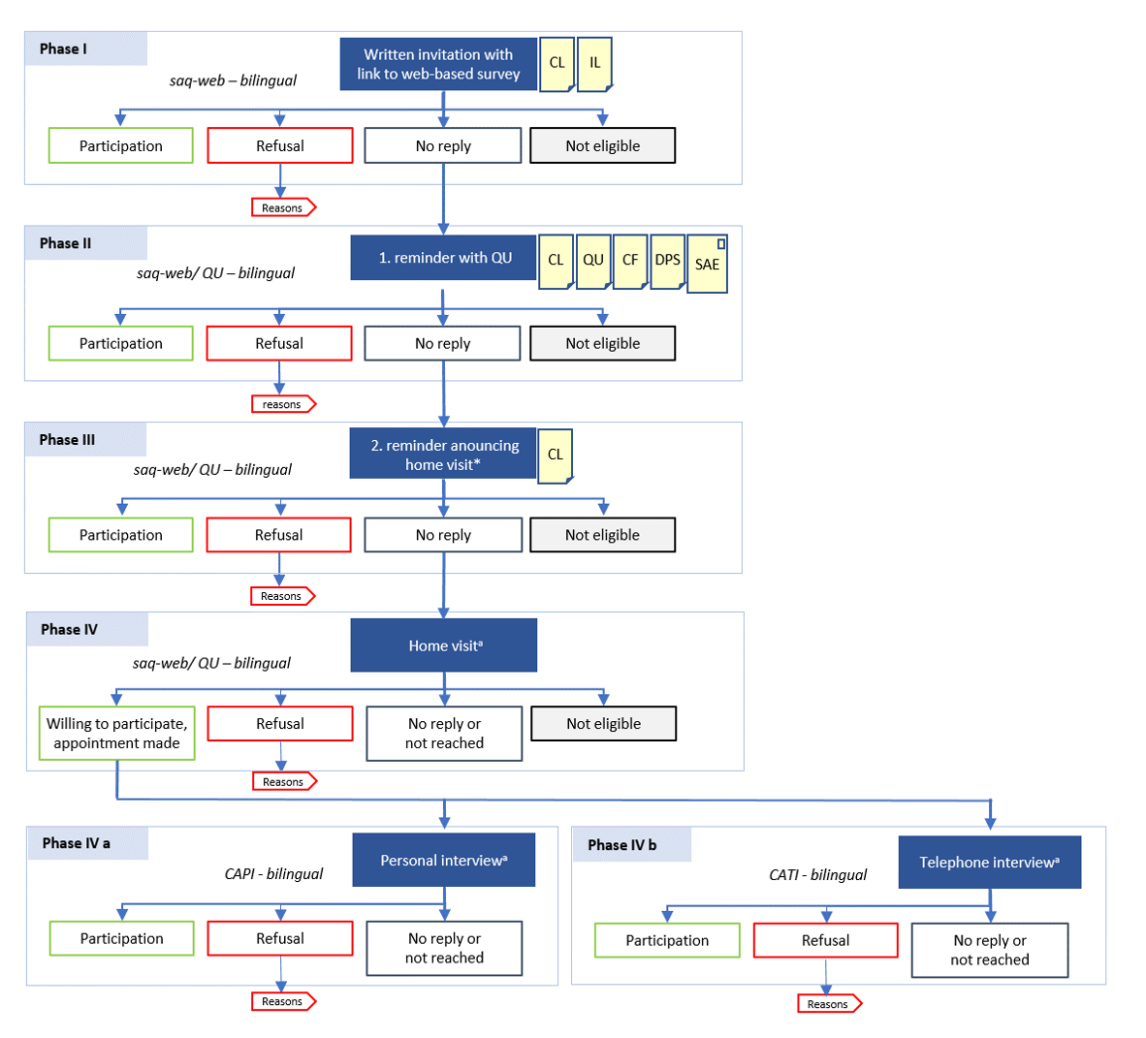
Study flow of the interview survey German Health Update Fokus (GEDA Fokus). CAPI: computer-assisted personal interview; CATI: computer-assisted telephone interview; CF: consent form; CL: cover letter; DPS: data privacy statement; IL: information leaflet; QU: paper-based questionnaire; SAE: self-addressed envelope.

If study persons refused participation, the reasons for nonparticipation were collected either on the telephone (hotline) or during personal contact at their home. The study flow is displayed in Figure 2. Data were collected from November 2021 to May 2022.

### Sample Size and Anticipated Response Rates

The calculated sample size was 1000 to 1200 participants per citizenship to enable stratified analyses by gender and 3 age groups for an expected prevalence of 5%. The anticipated response rate was 15% on average based on the experience of the feasibility study [23,24], resulting in a gross sample of 33,500 people.

Anticipated response rates were calculated per citizenship based on the response rates observed in the feasibility study (for web-based and personal interviews) and experiences from the GEDA survey 2014/2015 to account for the effect of the paper-based questionnaire on the response rate. The response rate of people with Italian citizenship was estimated as the mean of the response rates of the other citizenships. The response rate of people with Turkish citizenship was comparatively low in the feasibility study and was, therefore, corrected by calculating the mean of the response rate of the group with Turkish citizenship from the feasibility study and the estimated response rate of the group with Italian citizenship for GEDA Fokus. As phase IV in the feasibility study only took place among the groups with Syrian and Turkish citizenship, we estimated the effect of this study phase as the mean of both groups for the groups with Croatian, Italian, or Polish citizenship. We assumed a proportion of quality neutral losses of 10% in the PSUs with a BIK classification of <8 (n=46) and a proportion of 15% for the PSUs with a BIK classification of ≥8 (n=53). Table 1 summarizes the results of these calculations.

**Table 1:** Anticipated response rates and number of cases of the interview survey German Health Update Fokus (GEDA Fokus), stratified by Beratung Information Kommunikation (BIK) classification and study phases.

Citizenship	Anticipated response rates, %		Gross sample (unadjusted^a^), n		Gross sample (adjusted^b^), n		Participants (net sample), n			
	Phases I-III	Phase IV	BIK<8	BIK≥8	BIK<8	BIK≥8	Phases I-III		Phase IV	Total
							BIK<8	BIK≥8	BIK≥8	
Italian	15.23	5.85	867	5893	788	5125	120	780	300	1200
Croatian	14.3	5.85	923	6164	839	5360	120	766	314	1200
Polish	19	5.85	695	4998	632	4346	120	826	254	1200
Syrian	20.6	7.4	641	4436	583	3857	120	795	285	1200
Turkish	11.11	5.08	1188	7673	1080	6672	120	741	339	1200

^a^Includes the assumed proportion of quality neutral losses.

^b^Excludes the assumed proportion of quality neutral losses.

### Measurements

Key aspects of the survey are the core indicators for describing the health status of people with a migration background that were developed within the IMIRA project [25]. We added several social determinants of health, additional aspects of the utilization of health care, indicators for describing the impact of the COVID-19 pandemic, and the utilization of testing and vaccination. The approximated time for completing the questionnaire was 30 to 45 minutes. Textbox 1 lists the topics covered in the questionnaire.

**Topics covered within the German Health Update Fokus (GEDA Fokus) questionnaire.**

**Personal information**

- Age
- Sex and gender

**Overall health status**

- General health status
- Impairments in daily life
- Chronic diseases

**Diseases**

- Myocardial infarction
- Cardiac insufficiency
- Hypertension
- Asthma
- Tuberculosis
- Autoimmune disease
- Chronic kidney disease
- Rheumatoid arthritis
- Lipometabolic disorder
- Depression
- Anxiety disorder
- Coronary heart disease
- Stroke
- Diabetes
- Obesity
- Chronic bronchitis, chronic obstructive pulmonary disease, and emphysema
- Allergies
- Chronic liver disease
- Arthrosis or degenerative joint disease
- Chronic back pain
- Cancer

**SARS-CoV-2 or COVID-19**

- Laboratory test
- Hospitalization
- Effects of infection control measures
- Diagnosis
- Vaccination

**Health care**

- Hospitalization
- Utilization of therapy
- Need for health care services
- Vaccinations and utilization of medical services
- Utilization of general and specialist medical care
- Health insurance status
- Incapacity to work due to illness
- Care

**Living conditions**

- Social support and contacts
- Self-efficacy
- Subjective social status and life satisfaction
- Sense of belonging
- Self-perceived discrimination
- Religion and faith

**Health behavior**

- Physical activity
- Alcohol consumption
- Body weight and height
- Smoking
- Dietary intake
- Health information

**Migration status**

- Country of birth
- Reasons for migration
- Residence status
- Year of immigration
- Citizenship or citizenships
- Language proficiency (German and native language if other than German)

**Household and housing situation**

- Marital status
- Number of household members
- Type of building
- Size of living space

**Employment situation**

- Formal and vocational education
- Workplace exposure, shift work, and temporary employment
- Employment and occupational status
- Unemployment
- Income

### Weighting Procedures

A total of 2 weighting factors were calculated: one of them allows overall analyses across all 5 citizenship groups, and the other considers the marginal distributions within each citizenship group. On the one hand, the weighting factors control for the sampling of the PSUs, and on the other hand, they fit the sample to the marginal distribution of the population. When fitting the sample to the marginal distribution of the population, the weighting factors consider the distribution of region, gender, age, education (International Standard Classification of Education 2011), and the duration of residence of the 5 citizenship groups. Marginal distributions were taken from the Mikrocensus 2018 [27] after restricting the data to the 5 citizenship groups (including dual citizens).

### Quality Assurance

The translation of the questionnaire was done by 2 independent translators per language and was followed by a 2-stage editing process. In the first stage, an editor with a medical science background chose the better translations point by point. In the second stage, an editor without a medical scientific background proofread for general comprehensiveness. After typesetting (web-based and paper-based questionnaires), native speakers confirmed the correct translations as well as typesetting.

The data collection process was regularly monitored based on predefined indicators. The interviewers received training on the study aims and study design as well as on data protection and ethical aspects. Regular meetings for process evaluation took place, enabling the exchange of recruitment strategies between the interviewers and addressing the questions they raised.

### Ethics Approval

Ethics approval was received on December 22, 2021, from the ethics committee at the Medical University of Charité, Berlin (EA1/250/21). The study was approved by the Commissioner for Data Protection of the RKI without concern as of October 26, 2021. Written informed consent was obtained from all the study participants who completed a paper-based questionnaire or had a face-to-face interview. Oral consent was obtained from the study participants who had a telephone interview. Those who participated on the web consented by clicking on a respective button. The consent form was available in all 6 study languages. The interview data were anonymized and are stored at the RKI. After participation, all the study participants received an incentive in the form of a voucher worth €10 (US $11.4 as of January 3, 2022).

## Results

Overall¸ 6038 study participants took part in the survey, of whom 2983 (49.4%) were female. The median age was 39 years, and the median duration of residence in Germany was 10 years, with 19.69% (1189/6038) of the sample being born in Germany (unweighted). The characteristics of the study population stratified by citizenship group are displayed in Table 2. The overall response rate according to the American Association for Public Opinion Research (AAPOR; response rate 1) [28] was 18.4%, ranging from 13.8% in the group with Turkish citizenship to 23.9% in the group with Syrian citizenship. Furthermore, the response rates differed between the smaller municipalities where personal interviews were not offered (12.5%) and bigger cities where personal interviews were offered (19.3%). The self-administered web-based questionnaire (3028/6038, 50.15%) was the most opted mode of administration, followed by the paper-based version (1689/6038, 27.97%). Personal interviews in the study participants’ homes were conducted with 17.12% (1034/6038) of the participants, and telephone interviews were conducted with 4.75% (287/6038) of the participants. Of the 6038 participants, 2527 (41.85%) indicated that they had answered the questionnaire in German only, 1008 (16.69%) exclusively used the translation, and 2503 (41.45%) used both languages in the bilingual version of the questionnaire. More detailed results will be published soon.

**Table 2:** Description of the study population of the interview survey German Health Update Fokus (GEDA Fokus) (n=6038).

			Croatian (n=1223)				Italian (n=1205)				Polish (n=1193)				Syrian (n=1209)				Turkish (n=1208)		
			n (%)^a^		Weighted % (95% CI)		n (%)^a^		Weighted % (95% CI)		n (%)^a^		Weighted % (95% CI)		n (%)^a^	Weighted % (95% CI)			n (%)^a^		Weighted % (95% CI)
**Region**																					
	North	176 (14.39)		6.6 (2.4-16.7)		193 (16.02)		6.6 (2.9-14.4)		161 (13.5)		20.4 (9.5-38.6)		157 (12.99)			19.4 (9.8-34.7)	154 (12.75)		13.5 (6.0-27.5)	
	East	157 (12.84)		5.3 (1.5-16.8)		209 (17.34)		5.9 (1.9-17.1)		189 (15.84)		14.0 (4.1-38.3)		169 (13.98)			20.8 (7.4-46.1)	199 (16.47)		6.4 (1.6-21.9)	
	South	380 (31.07)		63.3 (50.1-74.9)		382 (31.7)		53.0 (40.1-65.5)		366 (30.68)		28.5 (18.6-41.0)		420 (34.74)			27.5 (17.4-40.6)	396 (32.78)		36.7 (25.3-49.8)	
	West	510 (41.7)		24.8 (15.7-37.0)		421 (34.94)		34.5 (23.7-47.2)		477 (39.98)		37.1 (24.8-51.3)		463 (38.3)			32.4 (21.2-46.1)	459 (38)		43.5 (30.7-57.2)	
**BIK^b^ classification**																					
	<8	125 (10.22)		29.7 (19.7-42.0)		148 (12.28)		36.2 (25.0-49.0)		134 (11.23)		26.0 (16.8-38.0)		148 (12.24)			35.9 (23.3-50.8)	152 (12.58)		19.3 (12.5-28.4)	
	≥8	1098 (89.78)		70.3 (58.0-80.3)		1057 (87.72)		63.8 (51.0-75.0)		1059 (88.77)		74.0 (62.0-83.3)		1061 (87.76)			64.1 (49.2-76.7)	1056 (87.42)		80.8 (71.6-87.5)	
**Sex**																					
	Female	663 (54.21)		47.7 (42.6-52.8)		581 (48.22)		40.5 (35.1-46.1)		685 (57.42)		51.4 (46.4-56.4)		759 (62.78)			64.6 (59.9-69.1)	604 (50)		49.0 (44.8-53.2)	
	Male	560 (45.79)		52.3 (47.2-57.4)		624 (51.78)		59.5 (53.9-64.9)		508 (42.58)		48.6 (43.6-53.7)		450 (37.22)			35.4 (30.9-40.1)	604 (50)		51.0 (46.8-55.2)	
**Age (years)**																					
	Median (range)^a^	42 (18-79)		N/A^c^		39 (18-79)		N/A		44 (18-79)		N/A		34 (18-79)			N/A	41 (18-79)		N/A	
	18-29	259 (21.18)		17.3 (13.3-22.2)		286 (23.73)		19.9 (16.0-24.4)		204 (17.1)		20.2 (15.9-25.2)		399 (33)			46.1 (40.1-52.3)	356 (29.47)		20.9 (17.6-24.6)	
	30-39	297 (24.28)		20.9 (16.8-25.6)		324 (26.89)		20.4 (15.4-26.4)		283 (23.72)		27.3 (23.4-31.5)		399 (33)			30.6 (26.5-35.0)	217 (17.96)		19.9 (16.0-24.4)	
	40-49	279 (22.81)		22.0 (17.9-26.7)		226 (18.76)		16.7 (12.7-21.7)		250 (20.96)		22.9 (19.3-27.0)		219 (18.11)			14.4 (11.1-18.5)	256 (21.19)		24.1 (20.6-28.0)	
	50-59	189 (15.45)		15.1 (11.0-20.4)		182 (15.1)		20.5 (15.8-26.1)		220 (18.44)		17.4 (13.8-21.8)		118 (9.76)			6.0 (4.5-7.9)	217 (17.96)		18.7 (16.0-21.8)	
	60-69	97 (7.93)		9.8 (7.0-13.7)		121 (10.04)		16.1 (12.0-21.2)		163 (13.66)		10.4 (7.7-13.9)		56 (4.63)			2.1 (1.4-3.2)	80 (6.62)		7.6 (5.5-10.5)	
	70-79	102 (8.34)		14.8 (10.3-20.9)		66 (5.48)		6.4 (4.4-9.4)		73 (6.12)		1.8 (1.3-2.5)		18 (1.49)			0.8 (0.4-1.6)	82 (6.79)		8.7 (6.1-12.4)	
**Education (ISCED^d^ 2011)**																					
	Low	255 (20.85)		29.8 (23.6-36.9)		252 (20.91)		44.7 (39.4-50.1)		239 (20.03)		19.8 (15.7-24.6)		452 (37.39)			54.2 (47.0-61.3)	506 (41.89)		62.9 (58.1-67.5)	
	Middle	592 (48.41)		56.4 (50.0-62.6)		413 (34.27)		39.2 (34.7-43.8)		500 (41.91)		55.7 (50.2-61.1)		385 (31.84)			28.6 (23.8-34.0)	370 (30.63)		30.6 (26.4-35.2)	
	High	373 (30.5)		13.8 (10.6-17.8)		536 (44.48)		16.2 (12.6-20.6)		447 (37.47)		24.5 (21.1-28.3)		364 (30.12)			17.2 (13.7-21.3)	322 (26.66)		6.5 (5.0-8.4)	
	Missing	3 (0.24)		N/A		4 (0.33)		N/A		7 (0.59)		N/A		8 (0.66)			N/A	10 (0.83)		N/A	
**Duration of residence (years)**																					
	Median (range)^a,e^	8 (1-79)		N/A		11 (1-68)		N/A		22 (1-67)		N/A		7 (1-64)			N/A	26 (1-74)		N/A	
	≤5	284 (23.22)		15.3 (11.4-20.2)		177 (14.69)		6.9 (4.5-10.5)		121 (10.14)		12.2 (9.2-16.0)		232 (19.19)			21.6 (16.8-27.2)	165 (13.66)		3.1 (1.9-5.0)	
	6-10	232 (18.97)		13.1 (9.8-17.4)		213 (17.68)		8.7 (6.0-12.5)		168 (14.08)		22.6 (17.3-28.8)		822 (67.99)			72.2 (66.2-77.4)	60 (4.97)		2.4 (1.4-4.1)	
	11-20	57 (4.66)		6.7 (4.1-10.7)		101 (8.38)		6.4 (4.1-10.0)		198 (16.6)		24.7 (19.8-30.2)		48 (3.97)			3.2 (1.8-5.9)	86 (7.12)		8.4 (5.8-12.0)	
	≥21	386 (31.56)		47.2 (39.9-54.6)		324 (26.89)		46.4 (40.8-52.0)		549 (46.02)		36.4 (30.4-42.8)		64 (5.29)			2.2 (1.2-4.2)	475 (39.32)		54.7 (50.1-59.3)	
	Since birth	251 (20.52)		17.7 (13.5-23.0)		381 (31.62)		31.6 (26.5-37.2)		125 (10.48)		4.2 (3.1-5.8)		36 (2.98)			0.8 (0.4-1.8)	396 (32.78)		31.4 (27.0-36.1)	
	Missing	13 (1.06)		N/A		9 (0.75)		N/A		32 (2.68)		N/A		7 (0.58)			N/A	26 (2.15)		N/A	

^a^Crude.

^b^BIK: Beratung Information Kommunikation.

^c^N/A: not applicable.

^d^ISCED: International Standard Classification of Education.

^e^Without the category “since birth.”

## Discussion

### Principal Findings and Outlook

We recruited the first nationwide sample of people with selected citizenships in Germany, representing the major groups with citizenships other than German. The sequential mixed-mode design and provision of multilingual study materials facilitated the participation of different groups of people in the study, for example, participants with lower proficiency in German or less preference for web-based participation. The data from this study will allow us to expand public health reporting on people with a history of migration in Germany, considering the core indicators developed within the scope of the IMIRA project [25]. Furthermore, we will be able to expand the knowledge on the impact of the COVID-19 pandemic on people with a history of migration. In addition, this sample will allow us to analyze factors associated with the health of people with a history of migration in a more differentiated manner, considering different migration-related and social determinants of health such as duration of residence, residence status, or living and working conditions.

### Limitations

First, sampling was only possible based on citizenship, as no other migration-related indicator is reliably captured within the residents’ registration offices. Therefore, not all groups of people with a history of migration are included, for example, naturalized people with only German citizenship or people with citizenships other than those that were selected. Therefore, conclusions cannot be drawn for all people with a history of migration living in Germany and can be drawn only for those with the 5 selected citizenships.

Sampling of the PSUs has some limitations, as we do not know the exact number of people with selected citizenships living within the municipalities and cities in Germany; this is especially the case for people with dual or multiple citizenships, as they are not captured within the Foreigners’ Statistics. Therefore, sampling was based on the overall proportion of people without German citizenship within the rural and urban districts. It was not always possible to sample the required number of people per citizenship (gross sample) within the smaller municipalities; this is especially true for people with Italian or Croatian citizenship in the northern and eastern parts of Germany. We filled these gaps by oversampling within the PSUs with a BIK classification of ≥8. Furthermore, the original selection of PSUs was based on the presumption that one-third of the net sample could be recruited in the PSUs with a BIK classification of <8. Once we encountered the problem of drawing enough people for the gross sample within the smaller PSUs, we adjusted this goal to recruit 10% of the net sample within the PSUs with a BIK classification of <8. This might result in cluster effects toward larger cities, which might limit comparisons between the rural and urban areas. Furthermore, this leads to a higher variance of the weighting factors for the participants in the PSUs with a BIK classification of <8. A higher variance of the weighting factors is associated with a lower precision of the estimates.

Furthermore, we offered only 6 languages, whereas other languages might have been required for respondents, for example, Kurdish, which might have been preferable for some people with Turkish or Syrian citizenship. In addition, we originally planned to apply a team translation approach, where 2 independent translators would translate the questionnaire, which would be followed by a moderated process with an editor to find the most accurate translation. Instead, translations underwent a 2-stage editing process; however, it was not possible to manage the moderated adjudication process of the team translation approach under the COVID-19 pandemic circumstances and time constraints.

The COVID-19 pandemic might have impacted the willingness to participate in the survey, as the public opinion on this topic, on the RKI as a national public health institute, and on the measures taken to slow down the spread of the virus were quite controversial during the period of data collection. This might have deterred some study persons from taking part in the survey; others might not have participated because of the fear of infection during the personal interviews. We attempted to address this issue by offering telephone interviews; however, the pandemic situation might have caused selection bias.

### Strengths

Besides these limitations, we want to highlight some strengths of the interview survey GEDA Fokus. First, it resulted in the largest sample of people with their own or a familial history of migration in Germany up to now. Although the indicator “citizenship” has its limitations, as mentioned earlier, we have certain marginal distributions according to Mikrocensus for the people with the selected citizenships, which allow us to weight data to yield generalizable results for this defined group.

The multimodal survey design resulted in accessibility for different groups of people, for example, for older people preferring paper-based questionnaires. In addition, the home visits with personal interviews as the gold standard [29-34] increased participation as personal contact builds trust, and concerns could be addressed directly. Offering multilingual questionnaires facilitated participation for study persons with lower German language proficiency because of, for example, a shorter duration of residence [23,24], as was the case among the participants with Syrian citizenship. Further methodological analyses will contribute to the understanding of how certain hard-to-survey subgroups among people with a history of migration can be reached and will be published soon.

The most newly developed survey instruments underwent cognitive pretesting [35,36] in the 5 translation languages before their application in GEDA Fokus to ensure a common understanding and comprehension of the questions [16,37]. Migration-related concepts, as well as indicators on discrimination, feelings of belonging, and self-efficacy, in connection with other social determinants of health, for example, housing, living, and socioeconomic aspects, will provide important insights into the complex interdependencies between social determinants and migrant health. Reporting the core indicators on the health of people with a migration background [25] will deliver important data to inform public health intervention planning. The indicators used in GEDA Fokus were also used in the European Health Interview Survey (EHIS), which will enable comparisons of the health status with the general population in Germany.

### Conclusions

We recruited approximately 6000 participants with Croatian, Italian, Polish, Syrian, or Turkish citizenship (1200 per group) living all over Germany. Multimodal survey administration, including the provision of personal interviews and bilingual study materials, facilitated the participation of different subgroups of people in the survey, resulting in a heterogeneous sample of people with their own or a familial history of migration with selected citizenships. Future data analyses will provide insights into the complex interplay between migration and health, considering different migration-related, social, and structural determinants of health.

## Abbreviations

AAPOR: American Association for Public Opinion Research
BIK: Beratung Information Kommunikation
EHIS: European Health Interview Survey
GEDA: German Health Update (Gesundheit in Deutschland aktuell)
IMIRA: Improving Health Monitoring in Migrant Populations
PSU: primary sampling unit
RKI: Robert Koch Institute

## Data Availability

The data sets generated during this study are not publicly available because of data protection reasons but are available from the corresponding author upon reasonable request.
